# Altered triple network model connectivity is associated with cognitive function and depressive symptoms in older adults

**DOI:** 10.1002/alz.14493

**Published:** 2025-03-05

**Authors:** Antonija Kolobaric, Carmen Andreescu, Andrew R. Gerlach, Eldin Jašarević, Howard Aizenstein, Tharick A. Pascoal, Pamela C. L Ferreira, Bruna Bellaver, Chang Hyung Hong, Hyun Woong Roh, Yong Hyuk Cho, Sunhwa Hong, You Jin Nam, Bumhee Park, Dong Yun Lee, Narae Kim, Jin Wook Choi, Sang Joon Son, Helmet T. Karim

**Affiliations:** ^1^ Department of Psychiatry University of Pittsburgh School of Medicine Pittsburgh Pennsylvania USA; ^2^ Department of Obstetrics, Gynecology and Reproductive Sciences University of Pittsburgh School of Medicine Pittsburgh Pennsylvania USA; ^3^ Department of Computational and Systems Biology University of Pittsburgh School of Medicine Pittsburgh Pennsylvania USA; ^4^ Magee‐Womens Research Institute Pittsburgh Pennsylvania USA; ^5^ Department of Bioengineering University of Pittsburgh School of Medicine Pittsburgh Pennsylvania USA; ^6^ Department of Psychiatry Ajou University School of Medicine Suwon‐si Gyeonggi‐do Republic of Korea; ^7^ Department of Biomedical Informatics Ajou University School of Medicine Suwon‐si Gyeonggi‐do Republic of Korea; ^8^ Department of Radiology Ajou University School of Medicine Suwon‐si Gyeonggi‐do Republic of Korea

**Keywords:** Alzheimer's disease, depression, machine learning, resting state connectivity

## Abstract

**INTRODUCTION:**

Late‐life cognitive impairment and depression frequently co‐occur and share many symptoms. However, the specific neural and clinical factors contributing to both their common and distinct profiles in older adults remain unclear.

**METHODS:**

We investigated resting‐state correlates of cognitive and depressive symptoms in older adults (*n* = 248 and *n* = 95) using clinical, blood, and neuroimaging data. We computed a connectivity matrix across default mode, executive control, and salience networks. Cross‐validated elastic net regression identified features reflecting cognitive function and depressive symptoms. These features were validated on a held‐out dataset.

**RESULTS:**

We discovered that white matter hyperintensities and nine overlapping nodes spanning all three networks are associated with both cognitive function and depressive symptoms, including left amygdala, left hippocampus, and bilateral ventral tegmental area.

**DISCUSSION:**

Our findings reveal intertwined neural nodes influencing cognitive impairment and depressive symptoms in late life, offering insights into shared characteristics and potential therapeutic targets.

**Highlights:**

- Resting‐state neuroimaging markers are associated with symptoms of cognitive decline and late‐life depression.
- Symptom‐associated connectivity alterations were present across three major brain networks of interest, including the salience, default mode, and executive control networks.
- Some regions of interest are associated with both cognitive function and depressive symptoms, including the left amygdala, left hippocampus, and bilateral ventral tegmental area.

## 1 BACKGROUND

Dementia, a progressive major neurocognitive disorder (MNCD), afflicts > 50 million people worldwide.^1^, ^2^ It encompasses various cognitive symptoms, including perturbations to attention, executive function, memory, language, perception, and social cognition.^3^, ^4^ Concurrently, 30% to 50% of individuals with cognitive decline experience late‐life depression (LLD), a complex disorder characterized by dysphoria, cognitive impairment, anhedonia, and heightened suicide risk.^5^, ^6^ The co‐occurrence of depression and cognitive impairment, including mild cognitive impairment (MCI) and MNCD, entails a complex interplay that diminishes quality of life and increases the likelihood of progression from MCI to dementia.^7^, ^8^, ^9^, ^10^ The rising prevalence of MCI, MNCD, and depressive symptoms highlights the urgent need for novel treatment and prognostic approaches.^11^, ^12^

The symptom diversity in MCI, MNCD, and LLD includes cognitive, affective, and behavioral symptoms indicating structural and functional disruptions within and between interconnected brain networks.^13^, ^14^, ^15^, ^16^ There are unique and overlapping symptoms of LLD and MCI/MNCD, highlighting their intricate relationship.^3^, ^4^, ^13^, ^14^, ^15^, ^16^, ^17^, ^18^, ^19^, ^20^, ^21^ Research has identified three primary neural networks associated with these symptoms: the default mode network (DMN), executive control network (ECN), and salience network (SN).^13^, ^15^, ^22^, ^23^, ^24^, ^25^ Disruptions in these interconnected networks have led to the “triple network model,” which provides a comprehensive framework for understanding the cognitive, affective, and behavioral symptoms in these conditions.^26^ Briefly, the DMN is active during self‐referential processing and shows reduced activity during complex tasks, ECN is involved in cognition and executive function, while SN evaluates emotional valence and controls attention.^26^, ^27^, ^28^, ^29^, ^30^

Individuals with MCI and MNCD show changes in the DMN associated with cognitive decline, characterized by lower connectivity of key DMN nodes such as the precuneus and posterior cingulate cortex.^31^, ^32^ Conversely, LLD is often associated with DMN hyperconnectivity.^33^, ^34^, ^35^ In MCI, lower connectivity in the ECN is common, particularly in the left superior frontal gyrus and bilateral cingulate gyrus,^36^ although findings may differ for MNCD.^37^ Similarly, LLD is linked to lower ECN connectivity, particularly between the left dorsolateral prefrontal cortex and the dorsal anterior cingulate, and the bilateral inferior parietal cortices.^38^ Additionally, while those with MCI and MNCD generally exhibit higher SN connectivity,^31^ LLD is more commonly associated with lower SN connectivity.^39^, ^40^

Beyond triple network connectivity alterations, MCI, MNCD, and LLD are associated with several other factors. These conditions are associated with anxiety, apathy, cerebrovascular disease, advanced biological aging, and pro‐inflammatory changes.^41^, ^42^, ^43^, ^44^ MNCD, particularly Alzheimer's disease (AD), is often associated with pathological brain changes including amyloid beta (Aβ) deposition and white matter hyperintensities (WMHs), which are a biomarker of small vessel disease.^43^, ^45^ Further, the apolipoprotein E (*APOE*) gene and blood biomarkers such as plasma phosphorylated tau (p‐tau) can help assess AD risk.^43^, ^46^

Understanding the shared and distinct symptoms and neural components of MCI, MNCD, and LLD could enhance our understanding of these disorders. Previous studies leveraging machine learning models on various imaging modalities (structural magnetic resonance imaging [MRI], resting state functional MRI [fMRI], amyloid imaging) have discovered the critical role of the medial temporal lobe and plasma p‐tau in diagnosing MCI/MNCD, assessing cognitive decline, and predicting progression to dementia.^46^, ^47^, ^48^, ^49^, ^50^, ^51^, ^52^, ^53^ Additionally, research has used machine learning to predict LLD both as a standalone condition and in the context of neuropsychiatric symptoms within MCI/MNCD. These studies indicate that higher levels of depressive and psychiatric symptoms are predictive of conversion to dementia, with significant predictors including antidepressant use, age, caregiver‐perceived triggers, self‐rated social isolation, and poor health.^54^, ^55^, ^56^, ^57^, ^58^, ^59^

Much of the existing machine learning literature emphasizes optimizing diagnostic processes or predicting conversion to dementia, rather than identifying features that are modifiable and could inform potential interventions. While several studies have compared changes in individuals with multiple conditions versus those with only one,^6^, ^9^, ^60^, ^61^ the distinction between shared and distinct neural factors underlying cognitive and depressive symptoms in late life remains unclear. To address this gap, we used a universal network taxonomy approach and machine learning on a large, transdiagnostic sample of older adults. We discover, validate, and interpret factors predictive of cognitive function and depressive symptoms.

## 2 METHODS

### 2.1 Participants and study design

Participants were enrolled in the ongoing Biobank Innovations for Chronic Cerebrovascular Disease with Alzheimer's Disease Study (BICWALZS), part of the Korea Biobank Project and overseen by the Korea Disease Control and Prevention Agency.^62^ BICWALZS is registered as KCT0003391 in the Korean National Clinical Trial Registry and has received ethical approval from the institutional review board (AJOUIRB‐SUR‐2021‐038). This study acts as a biobank platform, actively involving memory clinics at five universities and a community geriatric mental health center to facilitate research on cognitive decline and dementia.

At the time of this analysis, BICWALZS had recruited 713 participants from six sites. Participants voluntarily enrolled through clinic visits, with some followed for up to 4 years, with each providing written informed consent. Exclusion criteria included severe neurological or medical conditions like Parkinson's disease, cerebral infarction, or organ failure.

**RESEARCH IN CONTEXT**

- **Systematic review**: The authors reviewed the literature using traditional sources, including PubMed, conference abstracts, and presentations. Previous research emphasizes the importance of understanding shared and distinct neural factors underlying late‐life cognitive impairment and depressive symptoms. However, few studies have comprehensively investigated resting‐state correlates in older adults using clinical, blood, and neuroimaging data.
- **Interpretation**: Our study fills this gap by analyzing connectivity patterns in addition to demographic, clinical, and blood biomarkers, in two cohorts of older adults, identifying features associated with cognitive function and depressive symptoms. Our findings highlight overlapping neural nodes associated with both conditions, providing insights into potential therapeutic targets.
- **Future directions**: These results contribute to Alzheimer's disease and dementia research, guiding future investigations into targeted therapeutic interventions and offering avenues for further exploration of neural correlates in late‐life cognitive impairment and depressive symptoms.

We analyzed baseline data from 420 individuals sourced exclusively from sites capable of collecting neuroimaging, blood plasma, and gut microbiome data, namely a memory clinic at Ajou University Hospital and Suwon Community Geriatric Mental Health Center.

### 2.2 Neurological and psychiatric assessments

Participants received comprehensive psychiatric and neuropsychological evaluations.^63^ Subjective cognitive decline (SCD) was established in the absence of detected impairments according to the Clinical Dementia Rating (CDR)^64^ and Seoul Neuropsychological Screening Battery (SNSB).^65^ MCI was diagnosed based on a 0.5 CDR score and the expanded Mayo Criteria^66^ for MCI. Diagnosis of AD adhered to the core clinical probable AD criteria set forth by the National Institute on Aging–Alzheimer's Association,^67^ while vascular dementia diagnosis followed the major vascular neurocognitive disorder criteria.^68^ A psychiatrist conducted psychiatric assessments for all participants.

Current depressive symptoms were evaluated using the Korean version of the Montgomery–Asberg Depression Rating Scale (MADRS)^69^ and the Korean version of the Short Form of Geriatric Depression Scale (SGDS‐K).^70^ Measurement of general cognitive function was performed using the Mini‐Mental State Examination (MMSE).^71^ Anxiety symptoms were assessed using the South Korean version of Beck's Anxiety Inventory (KBAI).^72^

### 2.3 Blood collection and analysis

Blood samples were collected by venipuncture after an overnight fast in the morning. Informed consent was obtained from all participants regarding the collection and genotyping of blood genomic DNA. Genomic DNA was isolated from the blood samples, and single‐nucleotide polymorphism (SNP) genotyping was performed by DNA Link, Inc. (Seoul, Korea) using the Affymetrix Axiom KORV1.0‐96 Array (Thermo Fisher Scientific) according to the manufacturer's protocol. The *APOE* genotypes were derived from rs429358 and rs7412, which were included in the array. p‐tau217^+^ was quantified by ALZpath.^73^ Plasma Aβ_40_ and Aβ_42_ were evaluated using validated commercially available plasma assay (Quanterix).^74^

### 2.4 Neuroimaging data acquisition and processing

All participants underwent MRI using a GE DISCOVERY MR750w 3T scanner located at Ajou University Hospital. Participants were recruited through two pathways: direct hospital visits and referrals from the community mental health center.

Resting‐state blood‐oxygen‐level‐dependent (BOLD) images were obtained in a single band interleaved order with the following specifications: repetition time/echo time: 2000/30 msec, voxel size 1.88 × 1.88 mm with 4.5 mm slice thickness and 4.5 mm slice gap, 60° flip angle, a field of view of 128 × 128, and no acceleration. The acquisition consisted of 200 volumes, and the total scan duration was 30 minutes. During the resting‐state scan, participants were instructed to focus their gaze on a white crosshair displayed against a black background while remaining awake. A three‐dimensional (3D) magnetization‐prepared rapid gradient echo (MPRAGE) T1‐weighted (T1w) sequence and a T2w fluid‐attenuated inversion recovery (FLAIR) sequence are listed in Table S1 in supporting information.

### 2.5 Structural processing

Data were processed using Statistical Parametric Mapping Toolbox 12 (SPM12) in MATLAB, using fourth‐degree B‐spline interpolation and a normalized mutual information similarity metric. T2‐FLAIR images were co‐registered with MPRAGE before multispectral segmentation was performed segmenting the brain into six tissue types (e.g., gray/white matter). To address WMHs, we adjusted the number of Gaussian components to two for white matter, enhancing gray and white matter identification. This generates a deformation field that can be used to normalize functional imaging data. We then computed an automated intracranial volume (ICV) mask by threshold gray matter, white matter, and cerebrospinal fluid (CSF) probability maps by 0.1, conducted image filling and closing, and then computed a skull‐stripped MPRAGE.

### 2.6 WMH processing

We used an automated approach for WHM in T2w FLAIR images described previously.^75^ Cerebral and cerebellar white matter (WM) regions were segmented in the T1w image and mapped to T2w FLAIR space using SPM12^76^ and FreeSurfer^77^ version 7.1.1. *Z* transformed T2w FLAIR images (*Z*‐T2w FLAIR) were used to detect WMH. Given the rarity of cerebellar lesions in our dataset, we used the mean and standard deviation (SD) of cerebellar WM to *Z* transform the T2w FLAIR image. Voxels within the cerebral WM mask with a *Z* score of ≥ 2 in the *Z*‐T2w FLAIR image were classified as WMH. The total WMH volume (WMHV) was normalized by the ICV (WMHV = WMH/ICV) and log‐transformed for analysis.

### 2.7 Resting state preprocessing

Functional data were preprocessed with SPM12^76^ and in‐house MATLAB scripts (MathWorks). Preprocessing included: slice time correction using the temporally middle slice as the reference; motion correction via rigid body co‐registration to the mean; skull‐stripping using the brain extraction tool in FSL;^78^ co‐registration to the skull‐stripped MPRAGE, for which the mean functional image was used to calculate the affine transformation; normalization to Montreal Neurological Institute space using the structural deformation from structural processing with 2 mm isotropic resolution; and smoothing with an 8 mm full width at half‐maximum (FWHM) Gaussian kernel. We then conducted filtering by regressing out six motion parameters, five principal components of WM and CSF, and a series of sinusoids corresponding to unwanted frequencies outside of the band‐pass in resting state (i.e., a band‐pass filter 0.008–0.15 Hz). Finally, we flagged all participants with excessive motion, which was defined as > 20% of the volumes marked as spike artifacts defined as > 0.5 mm movements, as calculated by the ArtRepair toolbox.^79^

### 2.8 Seed‐based resting state connectivity

For each network of interest, we defined a core set of regions as described by Uddin et al.’s comprehensive review, which established a universal taxonomy of macro‐scale functional brain networks.^30^ These core regions were then mapped to the Automated Anatomical Labeling atlas 3 (AAL3).^80^ Because the definition of the ECN was less distinct compared to DMNs and SNs, we used seeds from both control and attention networks to define it. This resulted in 20 regions of interest (ROIs; Table S2 in supporting information). Given that the anatomical regions described by Uddin et al. did not match perfectly with those in the AAL3, some nodes were categorized as overlapping, meaning they were assigned to more than one network.

Using anatomical masks from AAL3, we extracted the average time series for each of the 20 ROIs unilaterally, resulting in 40 unilateral ROIs. We excluded five regions (ParaHippocampal_L, ParaHippocampal_R, Parietal_Sup_R, Thal_MDm_L, Thal_MDm_R) due to poor signal‐to‐noise ratios, which were defined as ≥ 5% of the subjects having a temporal signal to noise ratio (tSNR) < 40. To address high correlations between corresponding left and right hemisphere regions, we computed Pearson correlations and consolidated regions into bilateral categories when their correlation > 0.7. This process resulted in 24 regions of interest, including 13 unilateral and 11 bilateral regions (Table S3 in supporting information). Using these 24 ROIs, we computed an all‐to‐all connectivity matrix based on Pearson correlations, resulting in a 24 × 24 connectivity matrix, with 276 unique functional connectivity (FC) indices. All 276 unique FC indices were included in the machine‐learning models.

### 2.9 Statistical analysis

**FIGURE 1:**
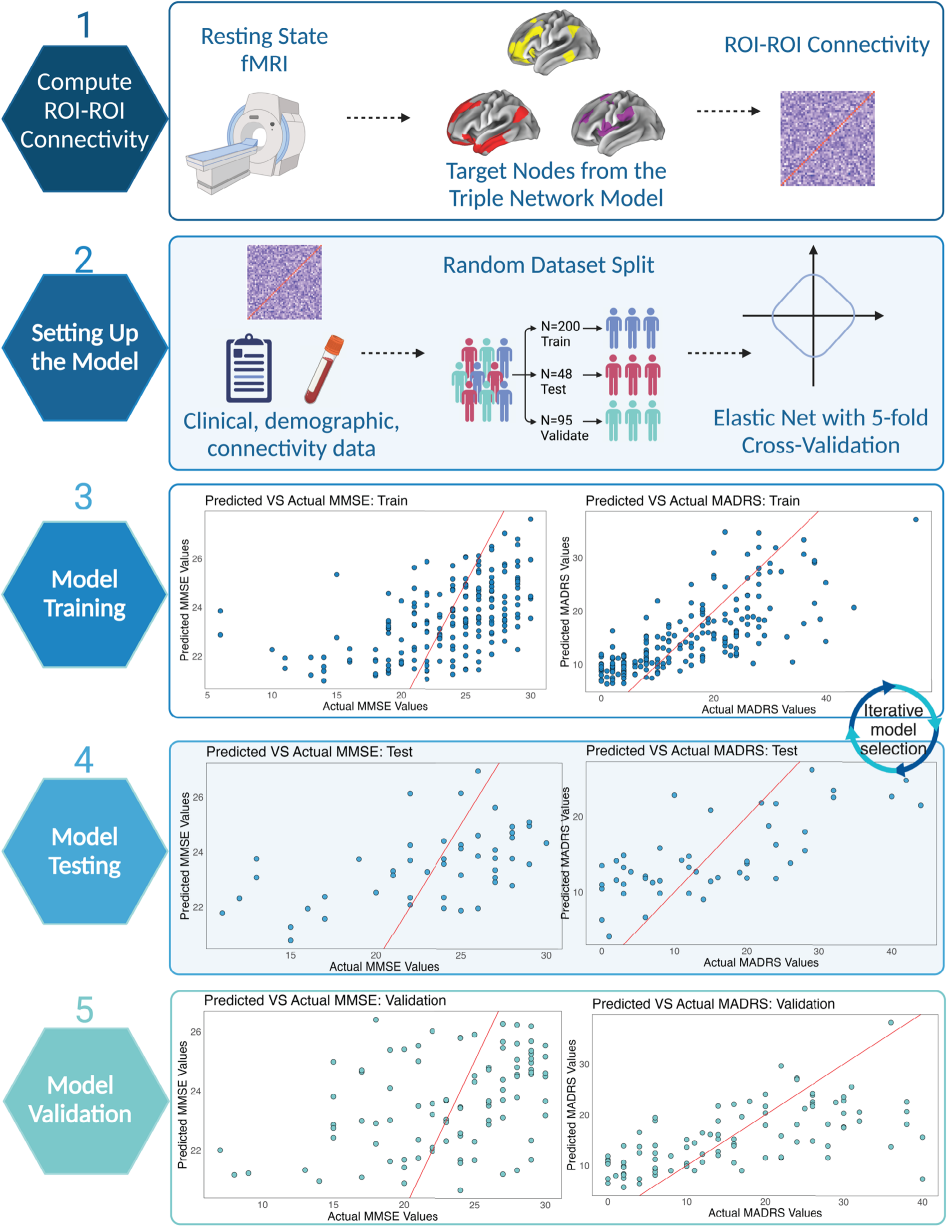
(Step 1) We chose target nodes from the default mode, executive control, and SNs and computed their ROI–ROI connectivity matrix based on resting‐state scans. (Step 2) We included 343 participants with high‐quality clinical, demographic, neuroimaging, and blood samples, dividing them into train, test, and validate datasets. The data was used in an elastic net model with 5‐fold cross‐validation and a grid search for hyperparameter optimization. (Step 3) Using the glmnet package in R, we performed two 5‐fold repeated cross‐validation procedures with 500 repetitions each, training the model and (Step 4) iteratively assessing its performance on the test dataset. (Step 5) The model was then validated using a separate validation dataset. The red line in steps 3 through 5 represents the idealized line of perfect agreement between actual and predicted values in the scatterplot. fMRI, functional magnetic resonance imaging; MADRS, Montgomery–Asberg Depression Rating Scale; MMSE, Mini‐Mental State Examination; ROI, region of interest; SNs, salience networks

Before conducting any statistical analyses, we implemented exclusion criteria, leading to the removal of 77 participants from our initial participant pool. These exclusions included 16 participants with primary psychiatric diagnoses other than depression, 21 with inadequate or missing MRI, 9 due to high motion during the scan as defined above, and 31 participants with incomplete or missing clinical data. As a result, our final dataset comprised 343 participants (Figure 1, Table 1, and Table S4 in supporting information).

**TABLE 1:** Participant sample summary at baseline: comparison between the train‐test and validate samples.

		Train–test	Validate	
Variable	Category	Mean(SD)/*N*(%)	Mean(SD)/*N*(%)	*P* value
Sex	Female	180 (72.6)	71 (74.7)	0.789
	Male	68 (27.4)	24 (25.3)	
Age		72.6 (6.9)	71.5 (8.1)	0.283
Education (years)		7.5 (4.8)	7.9 (5.2)	0.555
MADRS		14.9 (11.3)	16.0 (11.5)	0.411
Antidepressant use	No	126 (50.8)	41 (43.2)	0.251
	Yes	122 (49.2)	54 (56.8)	
MMSE		23.5 (4.6)	23.3 (5.3)	0.835
KBAI		9.0 (10.2)	8.8 (10.3)	0.848
plasma pTau‐217		3.1 (1.7)	2.9 (3.1)	0.742
**plasma Aβ42**		6.5 (3.8)	4.5 (4.5)	**<0.001**
*APOE* ε4	No	182 (73.4)	65 (68.4)	0.434
	Yes	66 (26.6)	30 (31.6)	
Normalized WMH		0.0 (0.0)	0.0 (0.0)	0.260
Psychiatric Dx	None	58 (23.4)	24 (25.3)	0.871
	Major Dep	112 (45.2)	40 (42.1)	
	Minor Dep	78 (31.5)	31 (32.6)	
**Cognitive Dx**	SCD	16 (6.5)	10 (10.5)	**0.020**
	MCI	175 (70.6)	50 (52.6)	
	MNCD‐AD	39 (15.7)	24 (25.3)	
	MNCD Other	18 (7.3)	11 (11.6)	
**Site**	1	137 (55.2)	66 (69.5)	**0.023**
	2	111 (44.8)	29 (30.5)	

Abbreviations: AD, Alzheimer's disease; *APOE*, apolipoprotein E; Dx, Diagnosis; KBAI, South Korean version of Beck's Anxiety Inventory; MADRS, Montgomery–Asberg Depression Rating Scale; MCI, mild cognitive impairment; MMSE, Mini‐Mental State Examination; SCD, subjective cognitive decline; SD, standard deviation; Site 1, Ajou University Hospital; Site 2, Suwon Community Geriatric Mental Health Center; WMHs, white matter hyperintensity.

In this sample, 47.9% of participants with cognitive decline were diagnosed with major depressive disorder. This is on the higher end, but still in line with previous literature showing that 30% to 50% of those with AD have concurrent major depressive disorder.^5^ Of those with cognitive decline, 6.60% reported having severe depression, as measured by MADRS scores ≥ 35.

We used the caret package in R to eliminate zero and near‐zero variance variables, but we did not identify any. The final feature space included demographic (sex, age, years of education), clinical (MMSE, CDR, MADRS, SGDS‐K, antidepressant use, plasma p‐tau217, plasma Aβ42, *APOE* ε4 gene presence), and the ROI–ROI connectivity. Despite plasma Aβ42/Aβ40 being the potentially more informative biomarker, we chose plasma Aβ42 for this analysis because all participants had it available while only 43% of participants had the ratio. Our primary objective was to construct two distinct predictive models: one for predicting MADRS scores and another for MMSE scores. To achieve this, we excluded the target variable (MADRS or MMSE) and any variables measuring equivalent constructs (SGDS‐K for MADRS and CDR for MMSE) from the set of predictors. All other dataset variables were included in the model, including all clinical and demographic information, as well as the 276 FC indices.

We divided the dataset into two subsets: a train–test (*N* = 248) and a validation (*N* = 95) set, based on the availability of gut microbiome samples. This criterion was strategically used to facilitate the harmonious integration of resting‐state and gut microbiota data, laying the groundwork for a forthcoming comprehensive analysis. Model training exclusively took place on the train–test dataset. To ensure result reproducibility and consistency, we set the random seed before commencing any analyses.

The train–test dataset was further split into a training set, encompassing *N* = 200 subjects, and a test set, comprising *N* = 48 subjects, using an 80–20 split to iteratively refine our model selection and training processes. Ultimately, we executed two 5‐fold repeated cross‐validation procedures, each involving 500 repetitions, using the elastic net algorithm implemented through the glmnet package in R. These procedures were conducted separately to predict MMSE and MADRS scores. During the model selection and training phase, we optimized hyperparameters (alpha and lambda) using a systematic grid search.

We explored 10 different alpha values (ranging from 0 to 1) and 100 different lambda values (ranging from 0.0001 to 15), with the aim of minimizing root mean squared error (RMSE). Finally, once our models were fully developed and trained, we evaluated their performance on the previously held‐out validation dataset to assess their predictive capabilities (Figure 1). Standardized *β* coefficients were transformed by multiplying the unstandardized *B* value by the SD of the feature divided by the SD of the outcome. *R* ^2^ score (coefficient of determination) was calculated using the MLmetrics package in R.

After developing the primary models for predicting cognitive function and depressive symptoms, we extended our analysis by constructing three additional models. The first additional model aimed to predict MMSE scores using only connectivity features. We created two more models for predicting MADRS scores: one model used only connectivity features to predict MADRS scores; the other model replicated the clinical, demographic, and connectivity features used in the primary model but excluded antidepressant use to evaluate its impact on the model performance.

## 3 RESULTS

Table 1 and Table S4 provide an overview of the participants’ baseline clinical and demographic details. Within our cohort, we observed 26 individuals with SCD, 225 with MCI, 63 with MNCD due to AD, and 29 with MNCD due to other factors (e.g., frontotemporal, vascular, with Lewy bodies). In terms of psychiatric diagnoses, 82 participants had no psychiatric diagnosis, while 152 were primarily diagnosed with major depressive disorder, and 109 had a primary diagnosis of minor depressive disorder (Table 1, Table S4).

**FIGURE 2:**
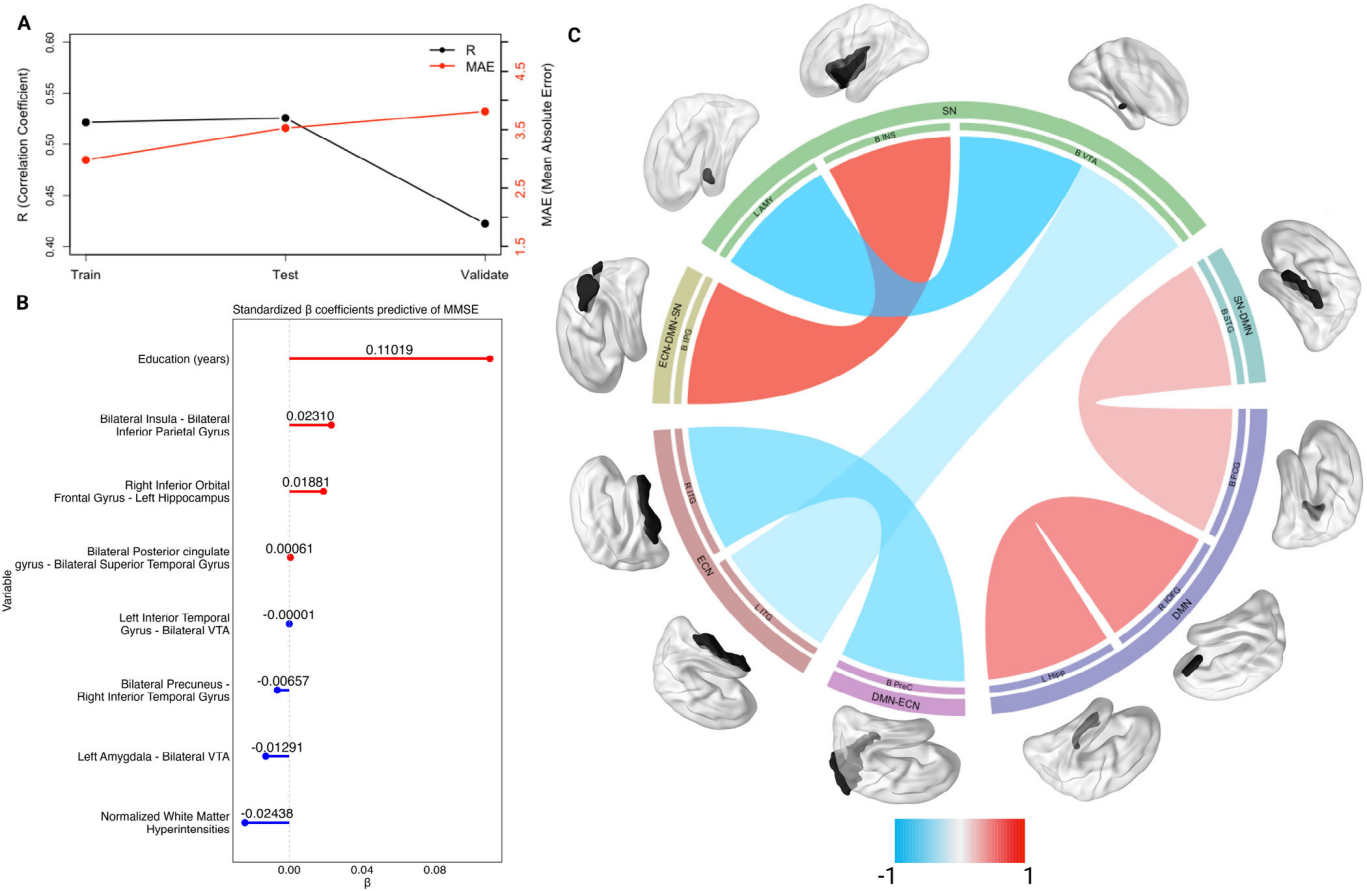
A, R and MAE and MSE values for the train, test, and validate datasets in the model predicting MMSE. B, Standardized coefficients from the elastic net regression used to predict MMSE scores. C, A circular plot demonstrating with and between network connectivity selected by the elastic net. Sample seeds used for the ROI–ROI connectivity are shown above the outer layer of the graph. B INS, bilateral insula; B IPG, bilateral inferior parietal gyrus; B PCG, bilateral posterior cingulate gyrus; B PreC, bilateral precuneus; B STG, bilateral superior temporal gyrus; B VTA, bilateral ventral tegmental area; B, bilateral; L AMY, left amygdala; L Hipp, left hippocampus; L ITG, left inferior temporal gyrus; L, left; MAE, mean absolute error; MMSE, Mini‐Mental State Examination; MSE, mean squared error; R IOFG, right inferior orbital frontal gyrus; R ITG, right inferior temporal gyrus; R, right; ROI, region of interest

The main MMSE model based on the complete feature set predicted MMSE in the training dataset with a correlation coefficient between actual and predicted MMSE of *r*(200) = 0.52, *R* ^2^ score = 0.23, mean absolute error (MAE) = 2.98, alpha = 1, and lambda = 0.606 (Table 2, Figure 2, Table S5 in supporting information). The model performed well on the test and validation datasets, with *r*(48) = 0.53, *R* ^2^ score = 0.20, MAE = 3.53, and *r*(95) = 0.42, *R* ^2^ score = 0.16, MAE = 3.81, respectively. Lower cognitive function (i.e., lower MMSE) was predicted by greater normalized WMHs and greater connectivity between left amygdala–bilateral ventral tegmental area (VTA), bilateral precuneus–right inferior temporal gyrus, and left inferior temporal gyrus–bilateral VTA. Greater cognitive function was predicted by greater years of education, and greater connectivity between bilateral posterior cingulate gyrus–bilateral superior temporal gyrus, right inferior orbital frontal gyrus–left hippocampus, and bilateral insula–bilateral inferior parietal gyrus.

**TABLE 2:** Predictors of cognitive function as measured by MMSE obtained from training an elastic net model using glmnet.

Variable	*β*
Education (years)	0.11019
Normalized WMHs	−0.02438
Left amygdala–bilateral VTA	−0.01291
Bilateral posterior cingulate gyrus–bilateral superior temporal gyrus	0.00061
Right inferior orbital frontal gyrus–left hippocampus	0.01881
Bilateral insula–bilateral inferior parietal gyrus	0.02310
Bilateral precuneus–right inferior temporal gyrus	−0.00657
Left inferior temporal gyrus–bilateral VTA	−0.00001

*Note*: *β* refers to unstandardized beta coefficients.

Abbreviations: MMSE, Mini‐Mental State Examination; VTA, ventral tegmental area; WMHs, white matter hyperintensities.

**FIGURE 3:**
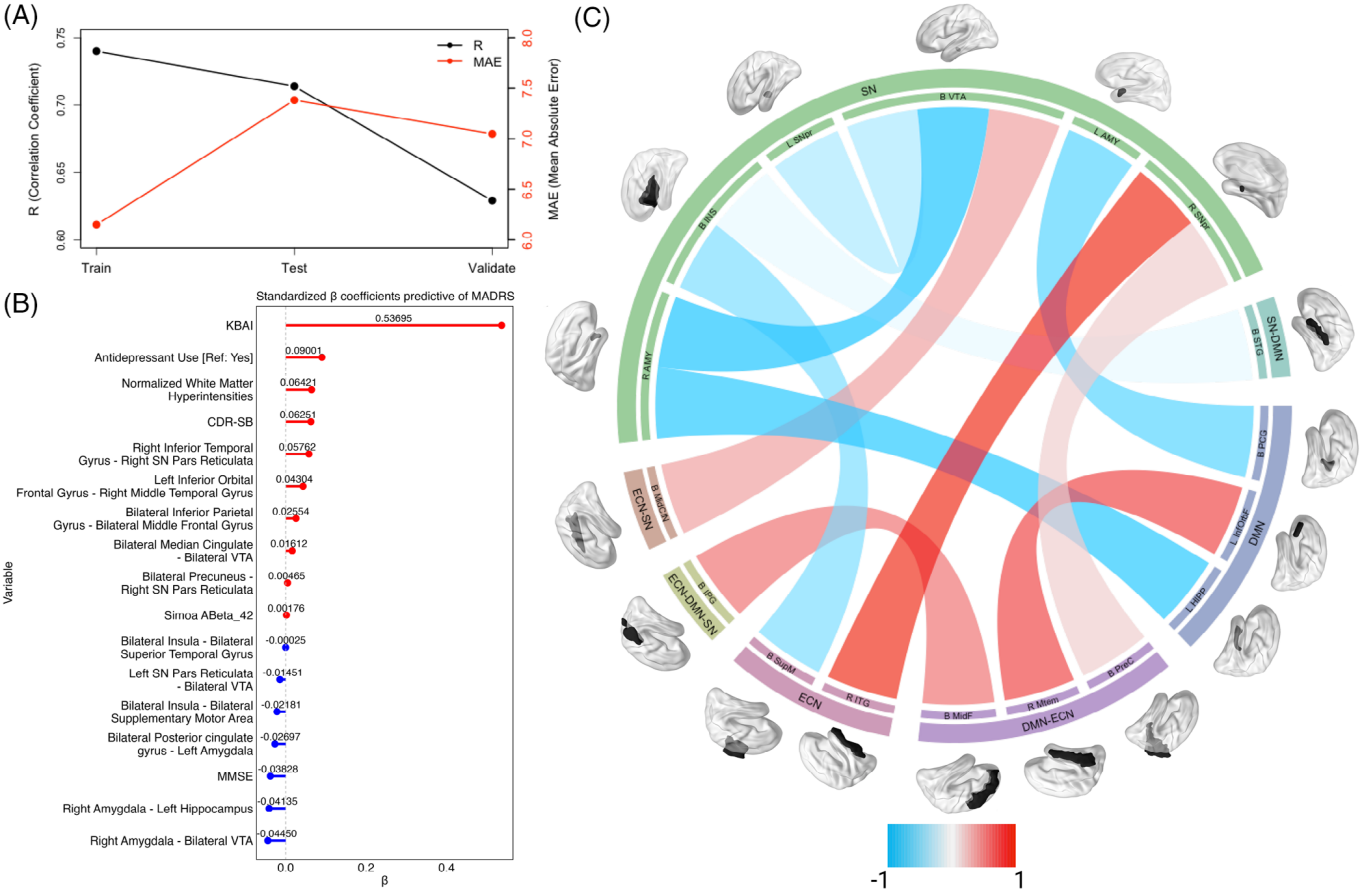
A, R and MAE and MSE values for the train, test, and validate datasets in the model predicting MADRS. B, Standardized coefficients from the elastic net regression used to predict MADRS scores. C, A circular plot demonstrating with and between network connectivity selected by the elastic net. Sample seeds used for the ROI–ROI connectivity are shown above the outer layer of the graph. B INS, bilateral insula; B IPG, bilateral inferior parietal gyrus; B MFG, bilateral middle frontal gyrus; B MidCIN, bilateral median cingulate; B PCG, bilateral posterior cingulate gyrus; B PreC, bilateral precuneus; B SMTA, bilateral supplementary motor area; B STG, bilateral superior temporal gyrus; B VTA, bilateral ventral tegmental area; B, bilateral; L AMY, left amygdala; L HIPP, left hippocampus; L IOFG, left inferior orbital frontal gyrus; L SNpr, left substantia nigra pars reticulata; L, left; MADRS, Montgomery–Asberg Depression Rating Scale; MAE, mean absolute error; MSE, mean squared error; R AMY, right amygdala; R ITG, right inferior temporal gyrus; R MTG, right middle temporal gyrus; R SNpr, right substantia nigra pars reticulata; R, right

The main MADRS model based on the complete feature set predicted MADRS in the training dataset with a correlation coefficient between actual and predicted MADRS of *r*(200) = 0.74, *R* ^2^ score = 0.52, MAE = 6.15, alpha = 1, and lambda = 0.909 (Table 3, Figure 3, Table S6 in supporting information). The model performed well on the test and validation sets, with *r*(48) = 0.71, *R* ^2^ score = 0.43, MAE = 7.38, and *r*(95) = 0.63, *R* ^2^ score = 0.38, MAE = 7.04, respectively. Greater depression (i.e., greater MADRS) was predicted by lower MMSE and lower connectivity between right amygdala–bilateral VTA, bilateral posterior cingulate gyrus–left amygdala, right amygdala–left hippocampus, bilateral insula–bilateral supplementary motor area, left substantia nigra pars reticulata (SNpr)–bilateral VTA, bilateral insula–bilateral superior temporal gyrus. Greater depression was predicted by greater CDR, KBAI, WMH normalized to head size (nWMH), plasma Aβ42, the use of antidepressants, and greater connectivity between bilateral precuneus–right SNpr, bilateral median cingulate–bilateral VTA, bilateral inferior parietal gyrus–bilateral middle frontal gyrus, left inferior orbital frontal gyrus–right middle temporal gyrus, right inferior temporal gyrus–right SNpr.

**TABLE 3:** Predictors of depressive symptoms as measured by MADRS obtained from training an elastic net model using glmnet.

Variable	*β*
CDR	0.06251
Antidepressant use (ref: yes)	0.09001
KBAI	0.53695
MMSE	−0.03828
Normalized WMHs	0.06421
Right amygdala–left hippocampus	−0.04135
Right amygdala–bilateral VTA	−0.04450
Bilateral median cingulate–bilateral VTA	0.01612
Bilateral posterior cingulate gyrus–left amygdala	−0.02697
Left inferior orbital frontal gyrus–right middle temporal gyrus	0.04304
Bilateral insula–bilateral supplementary motor area	−0.02181
Bilateral insula–bilateral superior temporal gyrus	−0.00025
Bilateral inferior parietal gyrus–bilateral middle frontal gyrus	0.02554
Bilateral precuneus–right SNpr	0.00465
Left SNpr–bilateral VTA	−0.01451
Right inferior temporal gyrus–right SNpr	0.05762
Plasma Aβ42	0.00176

*Note*: *β* refers to standardized beta coefficients.

Abbreviations: Aβ, amyloid beta; CDR, Clinical Dementia Rating; KBAI, South Korean version of Beck's Anxiety Inventory; MADRS, Montgomery–Asberg Depression Rating Scale; MMSE, Mini‐Mental State Examination; SNpr, substantia nigra pars reticulata; VTA, ventral tegmental area; WMHs, white matter hyperintensities.

**FIGURE 4:**
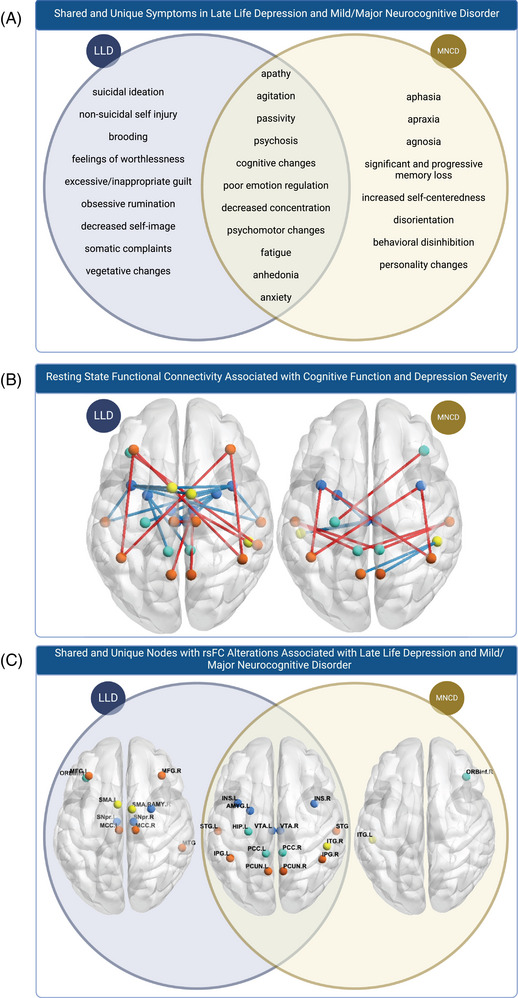
A, Overlapping symptoms between late‐life depression and mild or MNCD as described by previous research. B, rsFC linked to cognitive function and depression severity, based on elastic net results. Seed color indicates network belonging; blue: SN, cyan: DMN, orange: ECN, yellow: multiple networks. Red lines suggest higher connectivity was associated with higher MMSE/MADRS. Blue lines suggest lower connectivity was associated with lower MMSE/MADRS. C, Shared versus unique nodes with rsFC associated with cognitive function and depressive severity. DMN, default mode network; MADRS, Montgomery–Asberg Depression Rating Scale; MMSE, Mini‐Mental State Examination; MNCD, major neurocognitive disorder; rsFC, resting state functional connectivity; SN, salience network

Several features emerged as significant predictors of both MMSE and MADRS, including nWMH, and nine overlapping nodes: left amygdala, left hippocampus, right inferior temporal gyrus, bilateral inferior parietal gyrus, insula, posterior cingulate gyrus, precuneus, superior temporal gyrus, and VTA (Figure 4, Table S7 in supporting information). It is important to note that while we found nodes that appeared in pairwise resting state FC predictive of both cognitive function and depressive symptoms, none of the nodes formed the same connections between the two conditions (Figure 2, Figure 3).

Having examined models trained on the complete set of features, we next looked at the results of the three additional models we ran to further understand our results.

First, the model trained exclusively on connectivity features predicted MMSE in the training dataset with a correlation coefficient between actual and predicted MMSE of *r*(200) = 0.41, *R* ^2^ = 0.16, MAE = 3.41, alpha = 0.111, and lambda = 5.758. The model had the following results on the test and validation datasets, with *r*(48) = −0.21, *R* ^2^ = 0.04, MAE = 3.98, and *r*(95) = 0.14, *R* ^2^ = 0.02, MAE = 4.18, respectively.

The model trained exclusively on connectivity features predicted MADRS in the training dataset with a correlation coefficient between actual and predicted MADRS of *r*(200) = 0.39, *R* ^2^ = 0.15, MAE = 9.08, alpha = 0.222, and lambda = 7.576. The model had the following results on the test and validation datasets, with *r*(48) = 0.12, *R* ^2^ = 0.01, MAE = 9.60, and *r*(95) = –0.0017, *R* ^2^ = 0, MAE = 9.93, respectively.

Both models trained solely on connectivity data had similar patterns of performance—both models were able to capture some degree of relationship between predicted and actual scores but did so with modest correlation values and while explaining a small amount of variance. However, both models displayed a substantial drop in performance when applied to test and validation datasets, despite using 5‐fold cross‐validation. This suggests that, in the case of this particular dataset, the chosen connectivity features alone are not sufficient to predict cognitive performance or depressive symptoms.

In comparison, the model trained on all features except antidepressant use predicted MADRS in the training dataset with a correlation coefficient between actual and predicted MADRS of *r*(200) = 0.70, *R* ^2^ = 0.52, MAE = 6.46, alpha = 1, and lambda = 1.060. The model had the following results on the test and validation datasets, with *r*(48) = −0.76, *R* ^2^ = 0.58, MAE = 7.24, and *r*(95) = 0.61, *R* ^2^ = 0.37, MAE = 7.14, respectively. The predictive features were largely unchanged (Table S8 in supporting information). As the exclusion of antidepressants did not extensively impact model performance or selected features, we proceeded with the model that includes antidepressants as predictors for the remainder of the paper.

## 4 DISCUSSION

In this study, we set to identify shared versus distinct neural factors underlying cognitive and depressive symptoms in late life. We found that WMHs were highly predictive of both symptoms. While no region‐to‐region connectivity in the DMN, ECN, and SN were predictive of both cognitive and depressive symptoms, nine seeds (left amygdala, left hippocampus, right inferior temporal gyrus, bilateral inferior parietal gyrus, insula, posterior cingulate gyrus, precuneus, superior temporal gyrus, and VTA) were predictive of both cognitive and depressive symptoms in late life. Additionally, we identified features that correlated uniquely with either cognitive decline or depressive symptoms, highlighting the complexity of both depression and cognitive decline.

Greater WMH burden emerged as the strongest shared predictor, whereby greater burden was associated with worse cognitive function and higher depressive symptoms. Given the role of WMHs in cerebral small vessel disease and the effects on executive function, this finding aligns with prior research linking vascular disease to both LLD and cognitive dysfunction, potentially through the white matter dysconnectivity hypothesis.^81^, ^82^, ^83^ This hypothesis suggests that disrupted white matter connectivity impairs communication among brain regions, contributing to functional decline.^84^, ^85^ Additionally, notable sex differences exist in WMH progression, risk factors, and related impairments, with women generally showing greater WMH volumes and stronger links to cognitive decline.^86^, ^87^ Given our predominantly female sample, these sex‐specific effects may have enhanced the predictive power of WMH for cognitive and depressive symptoms in this cohort.

We did not find any shared region‐to‐region connectivity predictive of both depressive symptoms and cognitive function, despite the overlapping clinical features of MCI/MNCD and LLD. The absence of shared connectivity predictors furthers the idea that cognitive and depressive symptoms in late life, although frequently co‐occurring, may arise from distinct network interactions, rather than identical neural pathways. The relationship between MCI/MNCD and LLD is complex, with multiple pathways potentially associated with both conditions.^88^ While some models suggest direct causal links—positioning depression as either an independent risk factor, a prodrome, or a result of dementia—others emphasize indirect pathways involving brain alterations, genetic factors, and lifestyle influences.^6^, ^83^, ^88^, ^89^, ^90^, ^91^, ^92^, ^93^, ^94^ Both conditions exhibit considerable individual heterogeneity, suggesting distinct biological phenotypes, which may explain why we did not find common region‐to‐region connectivity predictive of depressive and cognitive symptoms. The absence of a unified connectivity pattern suggests that even in individuals diagnosed with both conditions, the neural underpinnings of cognitive impairment and depression may be functionally distinct, highlighting the importance of a dimensional approach that captures the variability in symptom expression. Shared features may also be obfuscated by the nature of our cohort, in which all participants have some degree of self‐reported cognitive decline, but only a subset show depressive symptoms. We might expect to see more overlap of salient features in a cohort that included participants without self‐reported cognitive decline.^95^, ^96^

Regardless of the underlying etiology, the co‐occurrence of cognitive decline and depressive symptoms significantly worsens quality of life and leads to poorer long‐term outcomes.^97^, ^98^, ^99^, ^100^ In light of this, our identification of nine distinct nodes associated with both cognitive and depressive domains is particularly important, as they may offer potential targets for improving these outcomes. We observed significant alterations in the posterior DMN, particularly in the posterior cingulate, precuneus, hippocampus, and bilateral inferior parietal gyrus. The posterior DMN is implicated in consciousness and memory processing, with DMN activity at rest essential for memory consolidation, often disrupted in MCI/MNCD.^31^, ^101^, ^102^ The posterior DMN is also vulnerable to early amyloid accumulation,^103^, ^104^ and altered connectivity in posterior DMN has been found to be unique to MCI with LLD, compared to just LLD.^105^ Our results support the hypothesis that posterior DMN alterations may be associated with depressive symptom–related cognitive changes.^106^

Within the ECN, we observed significant changes in the right inferior temporal gyrus, a region crucial for higher cognitive functions.^85^ This region has been shown to experience synaptic loss in both MCI and AD,^107^ and has previously been associated with depressive symptoms in normal aging.^108^ We also found significant alterations across the SN, including the left amygdala, bilateral insula, bilateral VTA, and bilateral superior temporal gyrus. The SN directs attention toward and away from stimuli, while also coordinating the DMN and ECN in response to stimulus presentation.^22^ We speculate that connectivity differences involving major SN regions could hinder the flexibility to transition between the ECN and DMN, a critical aspect of engaging executive functions like working memory and attention when confronted with cognitively demanding tasks,^109^, ^110^ which is a shared cognitive symptom for LLD and MCI/MNCD. However, while these regions may be commonly involved in both conditions, their connectivity patterns are context dependent, varying based on specific symptom profiles.

Additionally, our analysis revealed differences between the predictive models for cognitive and depressive symptoms. The MMSE model performed less effectively (*R* ^2^ = 0.18) on the held‐out validation dataset compared to the MADRS model (*R* ^2^ = 0.40). Furthermore, the MADRS model identified a greater number of predictive features. This discrepancy may be attributed to the nature of the measurements in this sample: the MADRS scores exhibited a higher coefficient of variation (CV = 74.53%) compared to the MMSE (CV = 20.58%), suggesting that the MADRS was able to capture the variance in depressive symptoms with more granularity than the MMSE, which is known to have ceiling effects.^111^

Moreover, the inclusion of anxiety and cognitive measures as predictors in the MADRS model likely enhanced its capacity to account for the heterogeneity of depressive symptoms. LLD is characterized by three primary facets: anhedonia–pessimism, anxiety–vegetative, and cognitive–inhibition.^112^ Having anxiety and cognitive measures as predictors captures two of three facets, likely leading to higher model performance. Consequently, the multifaceted nature of LLD, coupled with strong clinical predictors and the high variance of depressive symptoms in our sample, likely facilitated the MADRS model's improved performance and its ability to identify a greater number of predictive features compared to the MMSE model.

Clinically, our study identified WMHs and nine key nodes across the DMN, ECN, and SN as potential preventative or therapeutic targets. Interventions such as intensive blood pressure control may be successful in limiting the development of WMHs.^113^ Further, approaches such as transcranial magnetic stimulation (TMS) may offer a non‐invasive approach to modulate connectivity in the networks of interest. For example, in Figure 4C, there are a number of nodes that overlap between LLD and MNCD. In particular, the nodes of the precuneus, corresponding to the DMN, may be a potential target. One study showed that stimulation of the precuneus with 20 Herz repetitive TMS (2 weeks once daily combined with 22 weeks once weekly) in 25 patients with AD compared to 25 receiving sham, showed slowing of cognitive decline and neuropsychiatric symptom worsening.^114^ To date, TMS is determined to be safe in late life and has shown promise in LLD and cognitive decline.^115^, ^116^ Our findings identify potential hubs of networks, but more work is needed to identify the directionality of stimulation and their benefits. Our findings suggest that targeting nine key nodes may improve outcomes in those with coexisting cognitive and depressive symptoms, though future studies will be required to optimize target selection.

This study has several limitations. Our sampling was non‐probabilistic and limited to the South Korean population. Our sample is predominantly female and inherently biased toward individuals with subjective or diagnosed cognitive decline. Data from the two sites differed in plasma Aβ42 levels and diagnoses, likely due to one site recruiting from a hospital and the other from a geriatric community center. Including a wide range of diagnoses (SCD, MCI, MNCD) while relying on a single cognitive scale may overlook important illness‐specific information. Furthermore, we did not have information on the longevity of depression symptoms, which may impact our interpretation of the findings. Neuroimaging resolution may have introduced noise by capturing activity from neighboring regions in smaller nodes (e.g., SNpr, VTA), and the 7 minute resting‐state scan may affect the reliability of connectivity measures.^117^ We did not find that the connectivity alone was able to predict depressive symptoms or cognitive function in a generalizable way.

Our study investigated the associations between the resting state FC alterations associated with cognitive function and depressive symptoms in a large, transdiagnostic sample of older adults. As such, it representants an essential step forward at the intersection of reproducible machine learning and its applications to psychiatry, aging, and neuroimaging. Our results suggest that underlying functional connectivity alterations contribute to or reflect cognitive decline and depressive symptoms in late life, whereby the triple network model may be of particular interest. Future studies are needed to test if our model predicts future cognitive decline and depressive symptoms, potentially offering a biomarker for identifying people who may experience cognitive or mood decline. Such models would be of great benefit for treatment personalization, which may alter disease progression and increase quality of life in late life.

## AUTHOR CONTRIBUTIONS

Conceptualization/funding acquisition—Chang Hyung Hong, Hyun Woong Roh, Sang Joon Son; data generation—Chang Hyung Hong, Hyun Woong Roh, Yong Hyuk Cho, Sunhwa Hong, You Jin Nam, Bumhee Park, Dong Yun Lee, Narae Kim, Jin Wook Choi, Sang Joon Son; data curation—Antonija Kolobaric, Sang Joon Son; formal analysis—Antonija Kolobaric, Carmen Andreescu, Andrew R. Gerlach, Howard Aizenstein, Helmet T. Karim, Sang Joon Son; original draft—Antonija Kolobaric, Carmen Andreescu, Andrew R. Gerlach, Helmet T. Karim; and reviewing and editing—Antonija Kolobaric, Carmen Andreescu, Andrew R. Gerlach, Eldin Jašarević, Howard Aizenstein, Tharick A. Pascoal, Pamela C. L Ferreira, Chang Hyung Hong, Hyun Woong Roh, Yong Hyuk Cho, Sunhwa Hong, You Jin Nam, Bumhee Park, Dong Yun Lee, Narae Kim, Jin Wook Choi, Sang Joon Son, Helmet T. Karim.

## CONFLICT OF INTEREST STATEMENT

A.K. serves as a consultant for Radicle Science. C.A., A.R.G., E.J., H.A., T.A.P., P.C.L.F., C.H.H., H.W.R., Y.H.C., S.H., Y.J.N., B.P., D.Y.L., N.K., J.W.C., H.T.K., and S.J.S. report no biomedical financial interests or potential conflicts of interest. Author disclosures are available in the supporting information.

## CONSENT STATEMENT

Written informed consent was obtained from all participants and caregivers. BICWALZS is registered in the Korean National Clinical Trial Registry (KCT0003391) and approved by the institutional review board (AJOUIRB‐SUR‐2021‐038).

## ROLE OF THE FUNDER/SPONSOR

The funding sources had no role in the design and conduct of the study; collection, management, analysis, and interpretation of the data; preparation, review, or approval of the manuscript; or decision to submit the manuscript for publication.

## Supporting information



Supporting Information

Supporting Information

## Data Availability

Data and processing scripts are available from the corresponding authors upon reasonable request.
