# Supplementary material for: Altered triple network model connectivity is associated with cognitive function and depressive symptoms in older adults
Source: Alzheimers Dement. 2025 Mar 5;21(3):e14493. doi: 10.1002/alz.14493 (PMC11881620; doi:10.1002/alz.14493)
Supplement: Supplementary file 1 — Supporting Information [file ALZ-21-e14493-s001.docx]

# Title. Altered Triple Network Model Connectivity is Associated with Cognitive Function and Depressive Symptoms in Older Adults.

**Authors.** Antonija Kolobaric^1-4^, Carmen Andreescu^1^, Andrew R. Gerlach^1^, Eldin Jašarević^2-4^, Howard Aizenstein^1,5^, Tharick A. Pascoal^1^, Pamela C. L Ferreira^1^, Bruna Bellaver^1^, Chang Hyung Hong^6^, Hyun Woong Roh^6^, Yong Hyuk Cho^6^, Sunhwa Hong^6^, You Jin Nam^6^, Bumhee Park^7^, Dong Yun Lee^7^, Narae Kim^7^, Jin Wook Choi^8^, Sang Joon Son^6^*, Helmet T. Karim^1,5^*

**Affiliations.**

^1^Department of Psychiatry, University of Pittsburgh School of Medicine, 3811 O’Hara St. Pittsburgh, PA, 15213 United States

^2^Department of Obstetrics, Gynecology and Reproductive Sciences, University of Pittsburgh School of Medicine, 300 Halket Street Pittsburgh, PA 15213, United States

^3^Department of Computational and Systems Biology, University of Pittsburgh School of Medicine, 3420 Forbes Avenue Pittsburgh, PA 15213, United States

^4^Magee-Womens Research Institute, 204 Craft Ave, Pittsburgh, PA 15213, United States

^5^Department of Bioengineering, University of Pittsburgh School of Medicine, 300 Technology Dr, Pittsburgh, PA 15213, United States

^6^Department of Psychiatry, Ajou University School of Medicine, 206, World cup-ro, Yeongtong-gu, Suwon-si, Gyeonggi-do, 16499, Suwon, Republic of Korea

^7^Department of Biomedical Informatics, Ajou University School of Medicine, 206, World cup-ro, Yeongtong-gu, Suwon-si, Gyeonggi-do, 16499, Suwon, Republic of Korea

^8^Department of Radiology, Ajou University School of Medicine, 206, World cup-ro, Yeongtong-gu, Suwon-si, Gyeonggi-do, 16499, Suwon, Republic of Korea

* Co-corresponding authors

**Corresponding Authors:**

Sang Joon Son, MD

Department of Psychiatry, Ajou University School of Medicine

Suwon, Republic of Korea

E-mail: sjsonpsy@ajou.ac.kr

Helmet T. Karim, PhD

Department of Psychiatry and Bioengineering, University of Pittsburgh

Pittsburgh, PA, USA

E-mail: [hek26@pitt.edu](mailto:hek26@pitt.edu)

Supplemental Table S1. MRI acquisition parameters by sequence and recruitment site.

| **Sequence** | **Site** | **Acquisition matrix** | **Voxel size**  **(mm)** | **Repetition time (sec)** | **Echo time (msec)** | **Flip angle (⁰)** | **Slice thickness (mm)** |
| --- | --- | --- | --- | --- | --- | --- | --- |
| T1w MPRAGE | 1 | 256 x 256  512 x 512 | 0.39 x 0.39  0.78 x 0.78 | 7.1-9.9 | 2.8-4.8 | 12 | 1 |
|  | 2 | 256 x 256 | 1.00 x 1.00 | 7.4-7.6 | 2.7-2.8 | 11 | 1.2 |
| T2w FLAIR | 1 | 512 x 512 | 0.39 x 0.39 | 9.7 | 125 | 160 | 5 |
|  | 2 | 512 x 512 | 0.86 x 0.86 | 9 | 98 | 111 | 6 |
| fMRI | 1 | 128 x 128 | 1.875 x 1.875 | 2 | 30 | 60 | 4.5 |
|  | 2 | 128 x 128 | 1.875 x 1.875 | 2 | 30 | 60 | 4.5 |

Supplemental Table S2. The main hubs to define DMN, SN, and ECN. We chose main hubs for each network of interest as based on the Uddin 2019 paper. Next, we matched those regions with existing regions in the AAL3 atlas. The all-to-all connectivity between these features was used as an input to the elastic net models trained to predict MMSE or MADRS.

| **Default Mode Network** | | **Salience Network** | | **Executive Control Network** | |
| --- | --- | --- | --- | --- | --- |
| **Uddin Region** | **AAL3 Region** | **Uddin Region** | **AAL3 Region** | **Uddin Region** | **AAL3 Region** |
| medial prefrontal cortex | Frontal_Mid_2 | bilateral anterior insula | Insula | lateral prefrontal cortex along the middle frontal gyrus | Frontal_Mid_2 |
| posterior cingulate cortex | Cingulate_Post | anterior midcingulate cortex | Cingulate_Mid | anterior inferior parietal lobule, into the intraparietal sulcus. | Parietal_Inf |
| posterior extent of the inferior parietal lobule | Parietal_Inf | inferior parietal cortex | Parietal_Inf | midcingulate gyrus | Cingulate_Mid |
| inferior frontal gyrus, | Frontal_Mid_2 | right temporal parietal junction | Temporal_Sup | dorsal precuneus | Precuneus |
| middle temporal gyrus | Temporal_Mid | lateral prefrontal cortex | Frontal_Sup_Medial | posterior inferior temporal lobe, anterior to MT+ | Temporal_Inf |
| superior temporal sulcus | Temporal_Sup | substantia nigra/ventral tegmental area | VTA_Land SN_pr | dorsomedial thalamus and head of the caudate | Thal_MDm |
| parahippocampal cortex | ParaHippocampal | central nucleus of the amygdala | Amygdala | superior parietal lobule extending into the intraparietal sulcus | Parietal_Sup |
| areas dorsal and ventral to the posterior cingulate, the precuneus and retrosplenial cortex, respectively | Precuneus |  |  | middle temporal complex (MT+) | Temporal_Mid |
| hippocampus; | Hippocampus |  |  | the putative frontal eye fields (BA8) | Frontal_Mid_2 |
| superior/middle frontal gyrus | Frontal_Sup_2 |  |  | ventral premotor cortex. | Supp_Motor_Area |
| ventral frontal cortex | Frontal_Inf_Orb_2 |  |  | right-lateralized dorsolateral prefrontal cortex | Frontal_Sup_Medial |
| temporoparietal junction | Temporal_Sup |  |  |  |  |
| anterior temporal lobe | Temporal_Mid |  |  |  |  |

**Supplemental Table S3.** This table presents the classification of brain regions as either unilateral or bilateral, determined by measuring Pearson correlations between left and right hemisphere regions. When regions exhibited a correlation greater than 0.7, they were averaged and categorized as bilateral regions, while regions with correlations below this threshold remained classified as unilateral.

| **Bilateral (Combined)** | **Unilateral (L or R)** |
| --- | --- |
| Cingulate_Mid_B | Amygdala_L |
| Cingulate_Post_B | Amygdala_R |
| Frontal_Mid_2_B | Frontal_Inf_Orb_2_L |
| Frontal_Sup_2_B | Frontal_Inf_Orb_2_R |
| Frontal_Sup_Medial_B | Hippocampus_L |
| Insula_B | Hippocampus_R |
| Parietal_Inf_B | Parietal_Sup_L |
| Precuneus_B | SN_pr_L |
| Supp_Motor_Area_B | SN_pr_R |
| Temporal_Sup_B | Temporal_Inf_L |
| VTA_B | Temporal_Inf_R |
|  | Temporal_Mid_L |
|  | Temporal_Mid_R |

Supplemental Table S4. Participant sample summary at baseline. MADRS: Montgomery-Asberg Depression Rating Scale. MMSE: Mini Mental Status Examination. KBAI: South Korean version of Beck’s Anxiety Inventory. Dx: Diagnosis. SCD: Subjective Cognitive Decline. MCI: Mild Cognitive Impairment. MNCD: Major Neurocognitive Disorder. AD: Alzheimer’s Disease. Site 1: Ajou University Hospital. Site 2: Suwon Community Geriatric Mental Health Center.

| **Variable** | **Category** | **Mean(SD) / N(%)** |
| --- | --- | --- |
| Sex | Female | 251 (73.2) |
|  | Male | 92 (26.8) |
| Age |  | 72.3 (7.3) |
| Education (Years) |  | 7.6 (4.9) |
| MADRS |  | 15.2 (11.3) |
| Antidepressant Use | No | 167 (48.7) |
|  | Yes | 176 (51.3) |
| MMSE |  | 23.4 (4.8) |
| KBAI |  | 9.0 (10.2) |
| Plasma pTau-217 pg/mL |  | 3.0 (2.2) |
| Plasma Aβ42 |  | 5.9 (4.1) |
| APOE e4 | No | 247 (72.0) |
|  | Yes | 96 (28.0) |
| Normalized WMH |  | 8.37e-03 (9.66e-03) |
| Psychiatric Dx | None | 82 (23.9) |
|  | Major Dep | 152 (44.3) |
|  | Minor Dep | 109 (31.8) |
| Cognitive Dx | SCD | 26 (7.6) |
|  | MCI | 225 (65.6) |
|  | MNCD-AD | 63 (18.4) |
|  | MNCD Other | 29 (8.5) |
| Site | 1 | 203 (59.2) |
|  | 2 | 140 (40.8) |

**Supplemental Table S5.** Predictors of cognitive function as measured by MMSE (Mini Mental Status Examination) obtained from training an elastic net model using *glmnet*. B refers to unstandardized beta coefficients obtained directly form the *glmnet* results.

| **Variable** | **B Coefficient** |
| --- | --- |
| Normalized White Matter Hyperintensities | -32.73533 |
| Left Amygdala - Bilateral VTA | -0.60955 |
| Bilateral Precuneus - Right Inferior Temporal Gyrus | -0.33039 |
| Left Inferior Temporal Gyrus - Bilateral VTA | -0.00066 |
| Bilateral Posterior cingulate gyrus – Bilateral Superior Temporal Gyrus | 0.02743 |
| Education (years) | 0.26203 |
| Right Inferior Orbital Frontal Gyrus - Left Hippocampus | 0.84805 |
| Bilateral Insula - Bilateral Inferior Parietal Gyrus | 1.50141 |
| (Intercept) | 21.89976 |

**Supplemental Table S6.** Predictors of depressive symptoms as measured by MADRS (Montgomery-Asberg Depression Rating Scale) obtained from training an elastic net model using *glmnet*. B refers to unstandardized beta coefficients. MMSE: Mini Mental Status Examination. CDR: Clinical Dementia Rating. KBAI: South Korean version of Beck’s Anxiety Inventory.

| **Variable** | **B Coefficient** |
| --- | --- |
| Right Amygdala - Bilateral VTA | -2.17737 |
| Bilateral Posterior cingulate gyrus - Left Amygdala | -1.54414 |
| Right Amygdala - Left Hippocampus | -1.43160 |
| Bilateral Insula - Bilateral Supplementary Motor Area | -1.11323 |
| Left SNpr - Bilateral VTA | -0.71788 |
| MMSE | -0.09953 |
| Bilateral Insula - Bilateral Superior Temporal Gyrus | -0.01033 |
| plasma Aβ42 | 0.00507 |
| Bilateral Precuneus - Right SNpr | 0.27521 |
| CDR | 0.36405 |
| KBAI | 0.56750 |
| Bilateral Median Cingulate - Bilateral VTA | 0.87850 |
| Bilateral Inferior Parietal Gyrus - Bilateral Middle Frontal Gyrus | 1.35344 |
| Antidepressant Use (Ref: Yes) | 2.00666 |
| Left Inferior Orbital Frontal Gyrus - Right Middle Temporal Gyrus | 2.44240 |
| Right Inferior Temporal Gyrus - Right SNpr | 3.16941 |
| (Intercept) | 9.10730 |
| Normalized White Matter Hyperintensities | 88.12183 |

**Supplemental Table S7.** Shared predictors of depressive symptoms as measured by MADRS and cognitive function as measured by MMSE and their determined network affiliations.

| **Feature** | **Network** |
| --- | --- |
| left hippocampus | DMN |
| bilateral posterior cingulate gyrus | DMN |
| bilateral precuneus | DMN-ECN |
| right inferior temporal gyrus | ECN |
| bilateral inferior parietal gyrus | ECN-DMN-SN |
| left amygdala | SN |
| bilateral insula | SN |
| bilateral VTA | SN |
| bilateral superior temporal gyrus | SN-DMN |

**Supplemental Table S8.** Predictors of depressive symptoms as measured by MADRS (Montgomery-Asberg Depression Rating Scale) obtained from training an elastic net model using *glmnet* **without including antidepressant use as a feature**. B refers to unstandardized beta coefficients. MMSE: Mini Mental Status Examination. CDR: Clinical Dementia Rating. KBAI: South Korean version of Beck’s Anxiety Inventory.

| **Most predictive variables excluding antidepressant use** | **Standardized Beta** |
| --- | --- |
| KBAI | 0.5397 |
| CDR-SB | 0.0668 |
| Right Inferior Temporal Gyrus - Right SN Pars Reticulata | 0.0511 |
| Normalized White Matter Hyperintensities | 0.0479 |
| Bilateral Inferior Parietal Gyrus - Bilateral Middle Frontal Gyrus | 0.0186 |
| Bilateral Inferior Parietal Gyrus - Bilateral Middle Frontal Gyrus | 0.0168 |
| Bilateral Precuneus - Right SN Pars Reticulata | 0.0057 |
| Bilateral Median Cingulate - Bilateral VTA | 0.0008 |
| Left SN Pars Reticulata - Bilateral VTA | -0.0008 |
| Bilateral Posterior cingulate gyrus - Left Amygdala | -0.0118 |
| MMSE | -0.0152 |
| Bilateral Insula - Bilateral Supplementary Motor Area | -0.0180 |
| Years of Education | -0.0218 |
| Right Amygdala - Bilateral VTA | -0.0245 |
| Right Amygdala - Left Hippocampus | -0.0352 |
